# MiR‐17 family‐mediated regulation of Pknox1 influences hepatic steatosis and insulin signaling

**DOI:** 10.1111/jcmm.13902

**Published:** 2018-10-19

**Authors:** Dan Ye, Guohua Lou, Tianbao Zhang, Fengqin Dong, Yanning Liu

**Affiliations:** ^1^ Department of Endocrinology and Metabolism The First Affiliated Hospital of School of Medicine Zhejiang University Hangzhou China; ^2^ State Key Laboratory for Diagnosis and Treatment of Infectious Diseases Collaborative Innovation Center for Diagnosis and Treatament of Infectious Diseases The First Affiliated Hospital of School of Medicine Zhejiang University Hangzhou China

**Keywords:** hepatocyte steatosis, insulin resistance, metabolic disease, miR‐17 family, Pknox1

## Abstract

The aberrant expression of Pknox1 is associated with hepatic glucose and lipid dysmetabolism status of type 2 diabetes mellitus (T2DM) and nonalcoholic fatty liver disease (NAFLD). However, the underlying mechanism causing Pknox1 overexpression in this pathological status remains unclear. By using miRNA target prediction programs, we found that the 3′‐UTR of the Pknox1 mRNA sequence contains highly conserved target sites of miR‐17 family. In a rat model of streptozotocin and high‐fat diet‐induced T2DM and NAFLD complication, the increased hepatic expression of Pknox1 was consistent with decreased expressions of miR‐17 family, especially miR‐17 and miR‐20a. Furthermore, an inverse correlation was observed between Pknox1 and miR‐17 and miR‐20a in free fatty acids‐induced hepatocyte steatosis. Dual‐luciferase reporter assay further showed that Pknox1 was a valid target gene of miR‐17 family. The ectopic expression of miR‐17 or miR‐20a could markedly suppress Pknox1 expression in hepatocytes. MiR‐17 or miR‐20a overexpression also resulted in significantly enhanced insulin sensitivity and reduced hepatocyte steatosis in HepG2 and L02 cells, which were determined by altered phosphorylation on insulin receptor signaling pathway proteins and decreased intracellular triglyceride and lipid accumulation, respectively. These data implicate the upregulated hepatic expression of Pknox1 in T2DM complicated with NAFLD may be caused by the reduced expression of miR‐17 family, indicating that developing miRNA‐mediated regulation strategies on Pknox1 may provide new therapeutic options for metabolic disease.

## 1 INTRODUCTION

Nonalcoholic fatty liver disease (NAFLD) represents the most common liver disease in the whole world. Its development parallels that of insulin resistance (IR) and is associated with type 2 diabetes mellitus (T2DM).^1^ IR, in turn, facilitates excess liver fat deposition, oxidative hepatocellular damage, inflammation and activation of fibrogenesis, even progress toward cirrhosis and hepatocellular carcinoma. Thus, intrahepatocyte fat accumulation and systemic IR may influence with each other for leading to the progression of liver damage.^2^ However, the genetic bases of NAFLD and nonalcoholic steatohepatitis (NASH) risk and the relationship with IR are far from clear.

Transcription factor Pknox1 belongs to the three‐amino acid loop extension class of homeodomain proteins and is ubiquitously expressed both in fetal and adult mice and can form tripartite DNA‐binding complexes with members of the Hox and Pbx protein families.^3^ Pknox1 is essential at multiple stages of embryonic development.^4^ In mouse, Pknox1‐null embryos die before gastrulation, because epiblast cells undergo p53‐dependent apoptosis.^5^ Whereas, mouse embryos carrying a hypomorphic Pknox1^i/i^ mutation (expressing about 5% protein compared with WT) show a leaky embryonic‐lethal phenotype and defects in angiogenesis, hematopoiesis, and eye development.^4^, ^6^, ^7^ Thus, Pknox1 is previous known as a tumor suppressor associated with the maintenance of genomic stability. Recently, several studies have shown that Pknox1 was also involved in hepatic lipogenesis and insulin‐dependent glucose homeostasis.^8^ The Pknox1^i/i^ mice feature a complex phenotype characterized by increased insulin sensitivity and protection from streptozotocin (STZ)‐induced diabetes, accompanied with reduced hepatic lipogenesis and protection from methionine‐ and choline‐deficient diet (MCDD)‐induced steatohepatitis. While, hepatic expression of Pknox1 was significantly higher in the high‐fat diet (HFD)‐treated or T2DM (db/db) mice, suggesting aberrant Pknox1 expression may be associated with glucose and lipid dysmetabolism status of T2DM complicated with NAFLD.^9^ However, the underlying mechanism causing Pknox1 overexpression in this pathological status remains unclear.

MicroRNAs (miRNAs) are small noncoding RNAs that can cause mRNA degradation or translation inhibition by interacting with the 3′‐untranslated region (3′‐UTR) of the target gene mRNA.^10^, ^11^ Accumulated evidences have shown that miRNAs play crucial roles in the development of metabolic disease through regulating the expression of glucose and lipid metabolism and IR‐related genes.^12^ For example, miR‐122, as the predominant liver miRNA, has been proposed to play a central role in the maintenance of lipid and glucose homeostasis, it regulates hepatic fatty acid oxidation and cholesterol metabolism by way of downregulation of genes involved in cholesterol biosynthesis such as HMG‐CoA reductase^13^; miR‐149 decreases lipogenesis in hepatocytes by targeting fibroblast growth factor‐21 (FGF‐21) in the presence of free fatty acids (FFA) treatment^14^; miR‐34a inhibits very low density lipoprotein (VLDL) secretion and lipid accumulation, and improved hepatic steatosis in an HNF 4α‐dependent manner^15^; miR‐96 promotes the pathogenesis of hepatic IR through the suppression of insulin receptor (INSR) and insulin receptor substrate (IRS).^16^ However, certain miRNA, which can target and regulate the hepatic expression of Pknox1 has not been identified.

In this study, we used miRNA target prediction programs to explore Pknox1‐targeted miRNAs and identified miR‐17 seed family (miR‐17 family) as endogenous regulators of Pknox1. We then aimed to identify the role of miR‐17 family‐mediated regulation of Pknox1 in hepatic lipogenesis and IR, in order to seek new intervention targets for the prevention and treatment of progressive liver disease caused by T2DM and NAFLD complication.

## 2 METHODS

### 2.1 Rat model of T2DM complicated with NAFLD

Six‐week‐old male Wistar rats that weighed approximately 150 g were used for studies. The animals were reared under a specific pathogen‐free condition, and all procedures were reviewed and approved by the Institutional Animal Care and Use Committee of Zhejiang University. The induction of T2DM with NAFLD complication was performed according to Antony et al^17^ with some modifications. The rats were fed with HFD for 1 week and then intraperitoneally injected with 40 mg/kg STZ (Sigma‐Aldrich, St. Louis, MO, USA) after an overnight fast and fed with HFD until the end of the study. At the 2nd, 4th, and 8th week after STZ administration, the rats were killed and the blood and the liver samples were collected for subsequent analyses. Age‐matched rats were included as controls. For testing the insulin receptor signaling, mice were injected intraperitoneally with insulin solution (1 mU/g body weight) at 10 minutes prior to being killed.

### 2.2 Histological analysis

Formalin‐fixed, paraffin‐embedded specimens were cut into 8 μ mol L^−1^ serial sections and subjected to standard hematoxylin and eosin (H&E) staining for histological examination. HepG2 and L02 cells grown in 6‐well plates were washed with PBS and fixed with 10% neutral formalin, followed by staining with Oil Red O and hematoxylin. Sections and cells were imaged at 400× magnification (Olympus, Japan).

### 2.3 Biochemical analysis

Serum biochemical parameters including triglyceride (TG), alanine aminotransferase aspartate (ALT), aspartate transaminase (AST) levels were detected, using a Hitachi 7600 autoanalyzer (Hitachi, Tokyo, Japan), employing standard procedure. Blood glucose (GLU) was detected by Accu‐Chek Performa (Roche, Switzerland).

### 2.4 Cell culture and treatments

Human hepatic cell line HepG2 and L02 cells were grown in DMEM supplemented with 10% FBS and 1% penicillin/streptomycin in 5% CO_2_ at 37°C. In order to establish a cellular model of hepatic steatosis, cells were treated for 48 hours with a mixture of FFA including oleate and palmitate in a final ratio of 2:1, at a final concentration of 1 m mol L^−1^. To assess IR, IRS1, and ATK phosphorylation, cells were cultured with serum‐free DMEM for 12 hours, and then stimulated with insulin (200 n mol L^−1^) for 10 minutes.

### 2.5 Cellular triglyceride assay

Hepatocytes were collected for intracellular TG determination using a commercial kit (Applygen Technologies, China) according the manufacturer's instructions. TG values were normalized by total protein contents.

### 2.6 MiRNA analysis

For quantitative expression analysis of miRNAs, total RNAs enriched with miRNAs were isolated by mirVana™ miRNA Isolation Kit (Thermo fisher scientific, USA) from liver samples or HepG2 and L02 cells according to the manufacturer's instructions. Thereafter, cDNA was synthesized using PrimeScript^®^ RT reagent Kit (Takara, Japan) with specific miR‐17 family RT primers (RiboBio, China) according to the manufacturer's instructions. Relative quantitative real time PCR (qPCR) was performed, using the SYBR Premix Ex Taq kit (Takara) in the ABI Prism 7900 (Applied Bio systems, USA) with the primers of the above miRNAs (RiboBio). The comparative cycle threshold (Ct) method was applied to quantify the expression levels of miRNAs. Data are normalized over the average CT value of U6, and 2^−ΔΔCT^ method was used to determine relative miRNA expression.

### 2.7 Western blotting

Liver samples or hepatocyes were lysed with RIPA peptide lysis buffer (Beyotime Biotechnology, Jiangsu, China) containing 1% protease inhibitors (Pierce) and Western blotting analysis were performed according to standard procedures. Primary antibodies were used as follows: anti‐Pknox1 (1:1000, Novus, USA), anti‐IR (1:1000; Abcam, Cambridge, UK), anti‐pY‐IRS1 (1:1000; Abcam), anti‐IRS1 (1:2000; Abcam), anti‐pS‐IRS1 (1:2000; Abcam), anti‐pY‐IRS1 (1:1000; Abcam), anti‐AKT (1:1000; Cell Signaling Technology, MA, USA), anti‐pY‐AKT (1:1000; Cell Signaling Technology), and anti‐β‐actin (1:3000, Huabio, China). Protein bands were developed using the Enhanced Chemiluminescence (ECL) system and were visualized by using the ChemiScope Western Blot Imaging System (Clinx Science Instruments Co., Ltd). The gray‐scale value assay was performed by using Image J software (Rawak Software, Inc. Germany).

### 2.8 siRNA, miRNA and plasmid transfection

SiRNA against Pknox1 (si‐Pknox1) and scrambled siRNA (si‐Ctrl) were purchased from RiboBio. The mirVana miRNA mimics and inhibitors for miR‐17 and miR‐20a and the negative control (miR‐NC) were purchased from Ambion (Thermo fisher scientific). Pknox1 overexpression plasmid was constructed by Vigene biosciences. SiRNA and plasmid transfection was carried out using lipofectamine™ 3000 (Thermo fisher scientific), miRNA transfection was carried out using lipofectamine™ RNAiMAX (Thermo fisher scientific) according to the manufacturer's instructions. At 24 hours after transfection, the effects of siRNA‐ or miRNA‐mediated gene silencing and translational regulation were measured by western blot analysis.

### 2.9 Dual‐luciferase assay

The 3′‐UTR of Pknox1 containing the predicted miR‐17‐binding sites was amplified and subcloned into the pmiR‐RB‐REPORT™ vector to generate the Pknox1 Dual‐Luciferase expression vector (Pknox1‐WT). Mutations were performed, using a fast mutation kit (NEB, Canada) to generate Pknox1‐Mut1, Pknox1‐Mut2, and Pknox1‐Mut1+2. HEK293T cells were cotransfected with Pknox1‐WT or Pknox1‐Mut and miR‐17 or the control mimic. After 48 hours, the cells were lysed, and the firefly and renilla luciferase activities were measured with the Dual‐Luciferase Reporter Assay System (Promega, WI, USA). The results are presented as the ratio of renilla luciferase activity to firefly luciferase activity.

### 2.10 RNA isolation and real‐time PCR

Total RNA was isolated from cells using the Trizol reagent (Thermo fisher scientific) and reverse‐transcribed using a PrimeScriptTM RT reagent Kit with gDNA Eraser (Takara) according to the manufacturer's instructions. ACC, FASN, SREBP1c, SCD‐1, GPAM, AGPAT6, LPIN1, DGAT1, ATGL, HSL, MGL, and β‐actin mRNA expression was quantified by way of qPCR, using Power SYBR Green PCR Master MIX (Thermo fisher scientific) in an ABI Prism 7900 (Applied Biosystems) according to the manufacturer's instructions. Data are normalized over the average CT value of β‐actin, and 2^−ΔΔCT^ method was used to determine relative mRNA expressions.

### 2.11 Statistical analysis

Differences between groups were analyzed, using conventional Student's *t* test or ANOVA. Each experiment was repeated at least three times, and the data are presented as mean ± SD. A *P* < 0.05 was considered to be statistically significant.

## 3 RESULTS

### 3.1 Hepatic Pknox1 expression level is inversely correlated with miR‐17 family

**Figure 1:**
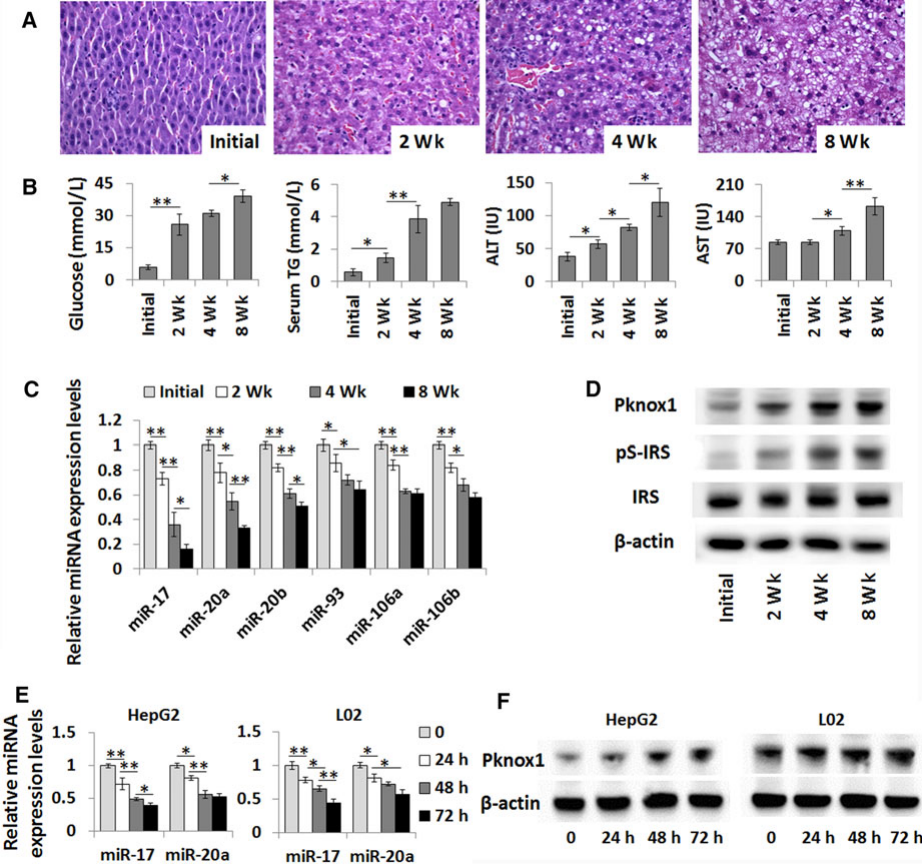
Pknox1 expression level is inversely correlated with miR‐17 family. A, Liver histology as determined by H&E. B, Blood GLU and serum TG, ALT, and AST contents were significantly elevated in the rat model of T2DM complicated with NAFLD. C, The hepatic expression levels of miR‐17 family were performed by real time‐PCR (qPCR). D, The protein levels of Pknox1, total IRS, and serine phosphorylation of IRS1 (pS‐IRS) in liver of the rat model were determined by Western blot analysis. E, The relative expression levels of miR‐17 and miR‐20a were performed by qPCR in FFA‐treated HepG2 and L02 cells. F, The protein levels of Pknox1 in FFA‐treated HepG2 and L02 cells were determined by Western blot analysis. Data are presented as the mean ± SD. (*P <* *0.05, **P <* *0.01, n = 3)

To address the significance of hepatic Pknox1 to the T2DM complicated with NAFLD, we first examined its expression in the rat model of STZ and HFD‐induced T2DM/NAFLD. Increased intrahepatic fat accumulation was confirmed by H&E staining of liver sections (Figure 1A). Consistent with the pathologic alteration, the serum GLU, TG, ALT, and AST levels and hepatic Pknox1 expression level were increased with the modeling time (Figure 1B,D). Moreover, the serine phosphorylation of the insulin receptor substrate 1 (IRS1) was significantly increased, indicating that insulin receptor signaling was also impaired in this model (Figure 1D). However, there were no significant differences in the levels of these metabolic parameters and hepatic PKNOX1 among the age‐matched control rats (8‐, 10‐, 14‐week‐old) with normal feeding (Figure S1). These data suggest that the increased hepatic Pknox1 may be specifically associated with the aberrant metabolism status in T2DM/NAFLD model.

Subsequently, by using three publicly available algorithms (TargetScan, miRanda, and PicTar), we found that the 3′‐UTR of the Pknox1 mRNA sequence contains two highly conserved target sites of miR‐17 family (Figure S2). We then examined the expression levels of miR‐17 family in the liver samples of the T2DM/NAFLD rats. As we expected, the hepatic expression levels of miR‐17 family, especially miR‐17 and miR‐20a, were decreased and inversely correlated with those of Pknox1 in this model (Figure 1C).

To test if high fat in vitro has effects on Pknox1 and miR‐17 family expression as that in T2DM/NAFLD rats, HepG2 and L02 cells were cultured by an FFA containing medium. The expression levels of miR‐17 and miR‐20a in HepG2 and L02 cells were downregulated by FFA treatment in a time‐dependent manner consistent with an increased expression of Pknox1 (Figure 1E,F). These data indicate that aberrant Pknox1 expression may be associated with dysregulated level of miR‐17 family in the pathological status of T2DM and NAFLD.

### 3.2 Pknox1 is a valid target of miR‐17 family

**Figure 2:**
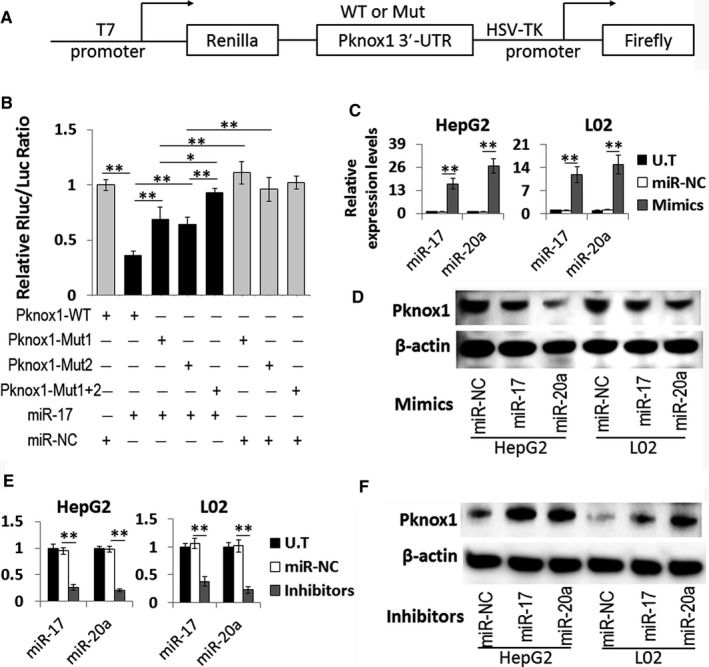
Validation of Pknox1 as the direct target of miR‐17 family. A, Wild‐type (WT) and mutant (Mut) 3′‐UTR of Pknox1 were cloned into a pmiR‐RBREPORT™ vector. B, Dual‐luciferase activity of the WT and Mut Pknox1 3′‐UTR reporter constructs in the presence of miR‐17or control miRNA (miR‐NC). Pknox1‐Mut1, Pknox1‐Mut2, and Pknox1‐Mut1 + 2 were represented as mutation of miR‐17‐targeted region 1 and 2 individually or of both regions in the Pknox1 3′‐UTR, respectively. (C and E) qPCR detection of miR‐17 and miR‐20a levels in HepG2 and L02 cells transfected with their mimics (C) or inhibitors (E) or the controls. (D and F) Western blot analysis of Pknox1 expression in HepG2 and L02 cells transfected with miR‐17 and miR‐20a mimics (D) or inhibitors (F) or the controls. Data are presented as the mean ± SD. (*P <* *0.05, **P <* *0.01, n = 3)

To test if Pknox1 is a true target of miR‐17 family, the 3′‐UTR fraction of Pknox1 containing the two predicted miR‐17‐binding sites was amplified and subcloned into the pmiR‐RB‐REPORT™ vector (Figure 2A). Then the luciferase construct (Pknox1‐WT) was transfected into HEK293T cells along with miR‐17 mimic, or a nontarget control miRNA (NC). Compared with miR‐NC, miR‐17 could induce a significant decrease in the normalized luciferase activity of the vector containing the putative miRNA‐binding site. While, mutation of either miRNA response element (MRE) individually (Pknox1‐Mut1 and Pknox1‐Mut2) or of both miR‐17‐targeted MREs in the Pknox1 3′‐UTR (Pknox1‐Mut1+2) resulted in partly or completely abrogation of the inhibitory effects of miR‐17 (Figure 2B). Similar results were found in the dual‐luciferase reporter assays on miR‐20a mimic and Pknox1‐WT or Pknox1‐Mut co‐transfected HEK293T cells (data not shown).

The regulation of Pknox1 expression by miR‐17 and miR‐20a was further verified by the transfection with their mimics and inhibitors. As anticipated, overexpression of miR‐17 and miR‐20a by transfection with their mimics both reduced Pknox1 protein levels in HepG2 and L02 cells (Figure 2C,D). On the contrary, by transfection with miR‐17 and miR‐20a inhibitors, the Pknox1 protein levels were increased accordingly (Figure 2E,F). These data strongly indicate that Pknox1 is a direct target of miR‐17 and miR‐20a. The upregulated expression of Pknox1 in the liver tissue of T2DM/NAFLD may be caused by the reduced expression of miR‐17 family.

### 3.3 MiR‐17 family‐mediated downregulation of Pknox1 enhances insulin receptor signaling in hepatocytes

**Figure 3:**
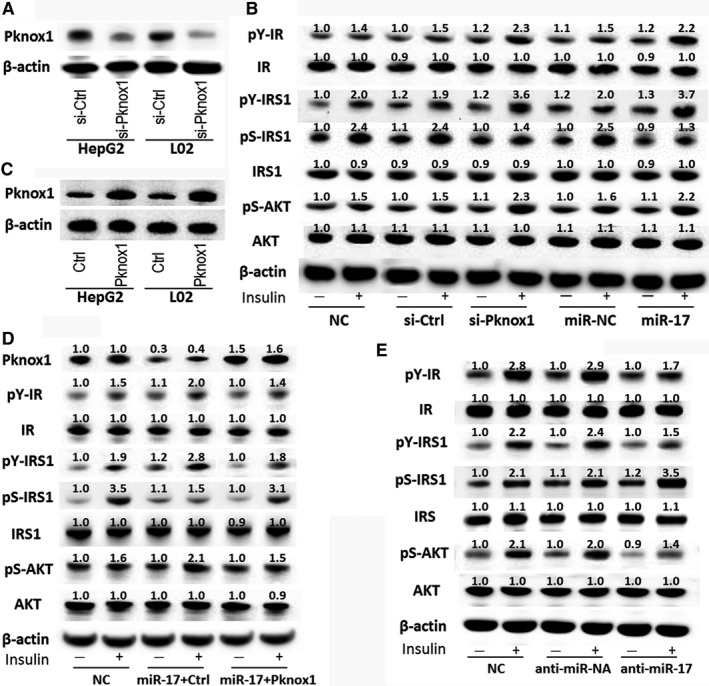
MiR‐17‐mediated regulation of Pknox1 affects insulin signaling in hepatocytes. A, Western blot analysis of Pknox1 expression in HepG2 and L02 cells transfected with Pknox1 siRNA (si‐Pknox1) or control siRNA (si‐Ctrl). B, HepG2 cells were transfected with si‐Pknox1 or miR‐17 mimic or their corresponding control for 48 h, then stimulated with insulin (200 n mol L^−1^) for 10 min. The IR, pY‐IR, IRS, pY‐IRS, pS‐IRS, AKT and pS‐AKT levels in HepG2 cells were determined by Western blot analysis. C, Western blot analysis of Pknox1 expression in HCC cells transfected with Pknox1 overexpression plasmid (Pknox1) or control plasmid (Ctrl). D, Restoration of Pknox1 level in HepG2 cells by transfection with Pknox1 plasmid reversed the effects of miR‐17 on the expressions of insulin signal transduction‐related genes. E, MiR‐17 inhibition impaired the insulin receptor signaling as determined by Western blot analysis of these genes. IR, insulin receptor; pY‐IR, Tyrosine phosphorylation of IR; IRS, insulin receptor substrate; pY‐IRS, Tyrosine phosphorylation of IRS; pS‐IRS, Serine phosphorylation of IRS1; pS‐AKT, Serine phosphorylation of AKT

Then we performed in vitro experiments to determine whether miR‐17‐mediated regulation of Pknox1 affects insulin signaling, similar to in vivo results in the T2DM/NAFLD rat model. After transfection with miR‐17 mimic or Pknox1 siRNA, HepG2 cells were then stimulated with insulin for 10 minutes. As shown in Figure 3B, downregulation of Pknox1 by transfection with Pknox1 siRNA (Figure 3A) or miR‐17 mimic reduced insulin‐induced serine phosphorylation of IRS1 whereas enhanced tyrosine phosphorylation of insulin receptor and IRS1 and the downstream phosphorylation of AKT, which all are known to be involved in insulin signal transduction.^9^, ^18^ However, co‐transfection of miR‐17 mimic with Pknox1 cDNA completely reversed the effect of miR‐17 mimic on insulin receptor signaling (Figure 3D). Moreover, transfection of miR‐17 inhibitor was shown to impair insulin receptor signaling as determined by affecting phosphorylation levels of these genes (Figure 3E). Similar results were found in miR‐20a mimic transfected L02 cells (data not shown). Together, these data suggest that miR‐17‐ or miR‐20a‐mediated suppression of Pknox1 regulates insulin action in hepatocytes.

### 3.4 MiR‐17 family‐mediated downregulation of Pknox1 reduces hepatocyte steatosis

**Figure 4:**
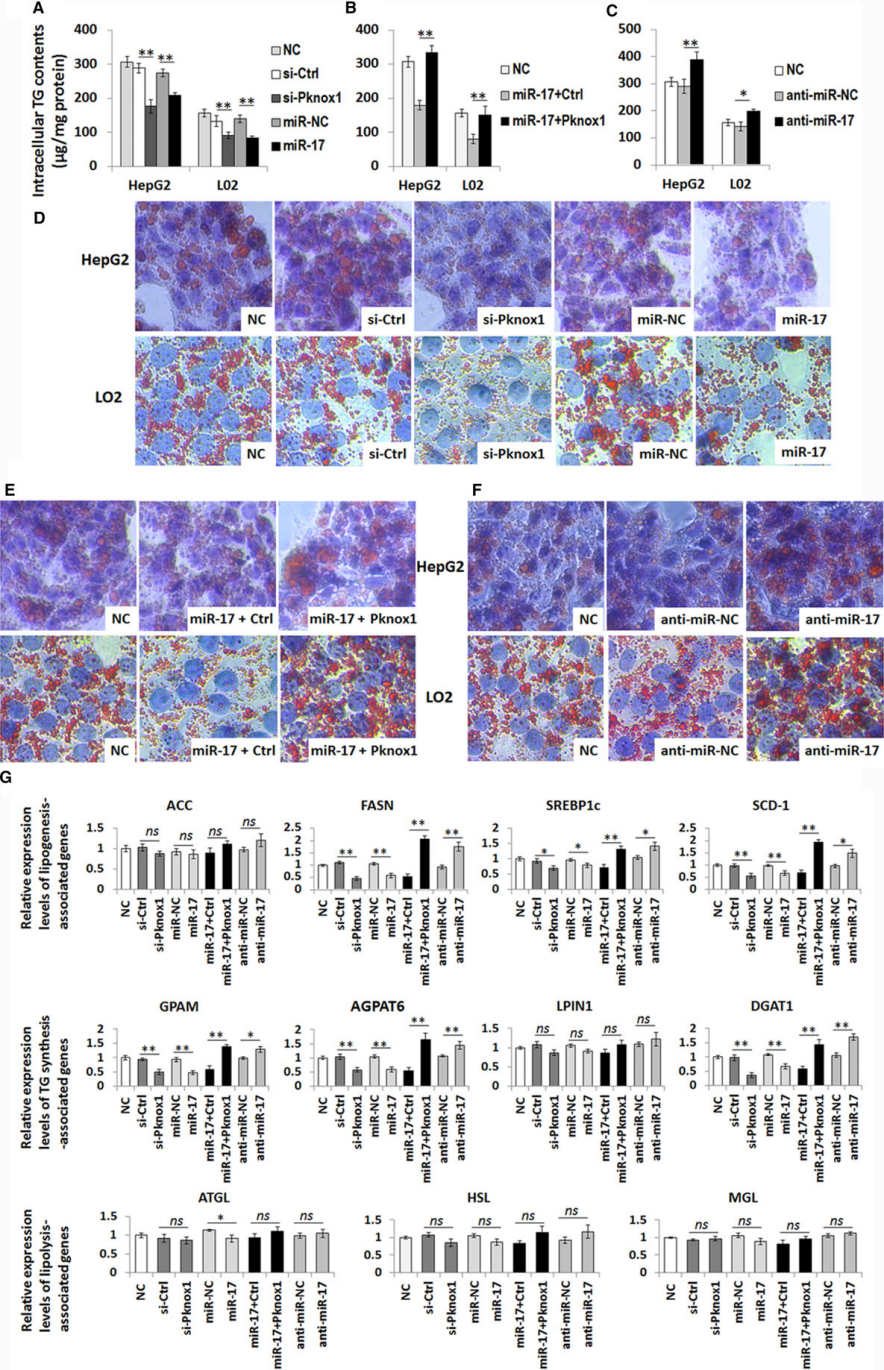
MiR‐17‐mediated regulation of Pknox1 affects hepatocyte steatosis. A, HepG2 and L02 cells were transfected with si‐Pknox1 or miR‐17 mimic or their corresponding control for 48 h, then exposed to FFA for 48 h. Intracellular TG contents in HepG2 and L02 cells were decreased by si‐Pknox1 or miR‐17 mimic transfection. B, Restoration of Pknox1 level in HepG2 and L02 cells by transfection with Pknox1 plasmid reversed the inhibitory effect of miR‐17 on TG content. C, MiR‐17 inhibition promoted lipogenesis in HepG2 and L02 cells. (D‐F) Representative image of Oil Red O staining of HepG2 cells corresponding to the above groups. G, Relative mRNA expression levels of genes involved in lipogenesis, TG synthesis, and lipolysis. Data are presented as the mean ± SD. (*P <* *0.05, **P <* *0.01, n = 3). ACC, acetyl‐CoA carboxylase; FASN, fatty acid synthase; SREBP1c, sterol regulatory element binding protein 1; SCD‐1, Stearoyl‐CoA desaturase; GPAM, glycerol‐3‐phosphate acyltransferase; AGPAT6, acylglycerol‐phosphate acyltransferase 6; LPIN1, lipin1; DGAT1, diacylglycerol acyltransferase 1; ATGL, adipose triglyceride lipase; HSL, hormone‐sensitive lipase; MGL, monoacylglycerol lipase

To further explore the impact of miR‐17‐mediated regulation of Pknox1 on hepatic steatosis, we transfected miR‐17 mimic or Pknox1 siRNA, or co‐transfected miR‐17 mimic with Pknox1 cDNA into HepG2 and L02 cells. As shown in Figure 4, downregulation of Pknox1 by transfection with Pknox1 siRNA or miR‐17 mimic decreased FFA‐induced intracellular TG (Figure 4A) and lipid accumulation in hepatocytes (Figure 4D). While, rescued Pknox1 expression by transiently transfection Pknox1 cDNA could revert the inhibitory effects of miR‐17 mimic on hepatocyte steatosis (Figure 4B,E). In contrast, transfection of miR‐17 inhibitor into HepG2 and L02 cells was shown to increase hepatocyte steatosis by FFA treatment (Figure 4C,F). In addition, the mRNA levels of genes involved in lipogenesis and TG synthesis were changed by miR‐17‐mediated Pknox1 regulation whereas those in lipolysis were not significantly altered (Figure 4G). Moreover, miR‐20a‐mediated regulation of Pknox1 showed similar effects on intracellular TG and lipid accumulation in HepG2 and L02 cells (data not shown). Overall, these results indicate that miR‐17 and miR‐20a reduce hepatocyte steatosis by targeting Pknox1.

## 4 DISCUSSION

Dysregulated expression of Pknox1 was shown to be associated with glucose and lipid dysmetabolism status of T2DM complicated with NAFLD.^9^, ^19^ In the rat model of STZ and HFD‐induced T2DM/NAFLD, we also found that the hepatic level of Pknox1 protein was significantly upregulated in consistent with the progression of hepatic steatosis and IR. However, the underlying mechanism leading to Pknox1 overexpression in this status is not well known. In this study, we identified miR‐17 family as a negative regulator of Pknox1 in hepatic fat accumulation and IR.

The miR‐17 family (also known as miR‐17‐92 cluster) maps to human chromosome 13 and consists of 6 individual miRNAs (miR‐17, miR‐18a, miR‐19a, miR‐19b, miR‐20a, and miR‐92a).^20^ The organization and sequences of the miR‐17‐92 family is highly conserved among vertebrates, and gene duplication and deletion events during evolution have resulted in two mammalian paralogs: the miR‐106b‐25 cluster and the miR‐106a‐363 cluster. The miRNAs encoded by miR‐17‐92 and its two paralogs can be grouped into four seed families (miR‐17, miR‐18, miR‐19 and miR‐92). Among them, miR‐17, miR‐20a, miR‐20b, miR‐93, miR‐106a, and miR‐106b belonged to the miR‐17 seed family, which share the same seed sequence.^21^ As shown in Figure S2A,B; the 3′‐UTR of the Pknox1 mRNA sequence contains two highly conserved target sites of miR‐17 seed family and one conserved target site of miR‐19 seed family. And it has been validated that Pknox1 as a target of both miR‐17 and miR‐19a in a previous study on mixed lineage leukemia.^22^ However, the hepatic miR‐19 level was slightly altered in the rat model of T2DM/NAFLD (Figure S2C). While, the hepatic levels of miR‐17 seed family, especially miR‐17 and miR‐20a, were significantly downregulated and inversely correlated with those of Pknox1 in this model. The inversed correlation between miR‐17 and Pknox1 was also confirmed in human samples (*r* = −0.981, *P *<* *0.01). We found that Pknox1 expression levels were increased in the liver sections of NAFLD patients as compared to those in the healthy subjects, especially in the patients complicated with T2DM. While, the expression levels of miR‐17 were decreased in the liver samples of NAFLD patient (Figure S3). Therefore, we focused on miR‐17‐ and miR‐20a‐mediated regulation of Pknox1 on hepatic glucose and lipid metabolism in the follow‐up experiments.

Besides miR‐17 family, miR‐223 was shown to target and inhibit Pknox1 gene expression.^23^ We also found that the hepatic level of miR‐223 was gradually decreased by HFD‐feeding and STZ administration. However, there is no obvious change of miR‐223 level in the hepatocytes cultured by FFA containing medium as compared to those by normal medium (Figure S4). It may be due to the specific expression tendency of miR‐223, which is preferentially expressed in the hematopoietic system and is considered to play important roles in the differentiation of hematopoietic stem cells, myeloid, erythroid and lymphoid cells.^24^ Thus, the dysregulation of miR‐223 maybe presented mainly in hepatic macrophage and affects its function by targeting Pknox1. The study by Zhuang et al. support and complement our speculation. They revealed that miR‐223 was a crucial regulator of macrophage polarization by way of inhibiting Pknox1 and then protected against diet‐induced adipose tissue inflammatory response and systemic IR.^23^ In contrast to macrophages, the expression of Pknox1 in hepatocytes was shown to be regulated mainly by miR‐17 family. In FFA‐induced hepatocyte steatosis, the hepatic expression level of Pknox1 was inversely correlated with miR‐17 and miR‐20a. In addition, mutation of either miR‐17 family‐targeted MREs or of both two MREs in the Pknox1 mRNA 3′‐UTR resulted in an increase in luciferase expression as compared to the wild‐type reporter construct, which further verified an indeed regulatory relationship between the miR‐17 family and the Pknox1 mRNA.

Moreover, supplement the level of miR‐17 or miR‐20a in hepatocytes by transfection with their mimics was shown to suppress Pknox1 expression and then enhanced insulin receptor signaling and reduced intracellular TG and lipid accumulation in hepatocytes. These observations indicate that it is possible that miR‐17 family mimics would serve as a novel approach for prevention and treatment of T2DM and NAFLD complication. However, to further determine the therapeutic effectiveness of miR‐17 family‐mediated regulation of Pknox1 in glucose and lipid metabolism disorders, the results from in vitro studies were verified in *in vivo* studies.

In conclusion, this study reveals that the downregulation of miR‐17 family may cause the aberrant Pknox1 expression in glucose and lipid dysmetabolism status of T2DM complicated with NAFLD. Furthermore, we provide evidence that upregulation of miR‐17 family can redress the insulin sensitivity and lipid metabolism in hepatocytes via targeting Pknox1. This evidence suggests a new pathogenic mechanism of NAFLD complicated with T2DM and may develop potential therapeutic strategies for metabolic disease.

## CONFLICT OF INTEREST

The authors declare that they have no competing interests.

## AUTHOR'S CONTRIBUTIONS

YD and LG performed the research and drafted the manuscript. ZT carried out the rat model and performed the in vivo studies. DF participated in *in vitro* studies and performed the statistical analysis. LY conceived of the study, and participated in its design and coordination. All authors read and approved the final manuscript.
