# Supplementary material for: MiR‐17 family‐mediated regulation of Pknox1 influences hepatic steatosis and insulin signaling
Source: J Cell Mol Med. 2018 Oct 19;22(12):6167–75. doi: 10.1111/jcmm.13902 (PMC6237553; doi:10.1111/jcmm.13902)
Supplement: Supplementary file 1 [file JCMM-22-6167-s001.docx]

**Supplementary Information:**

**
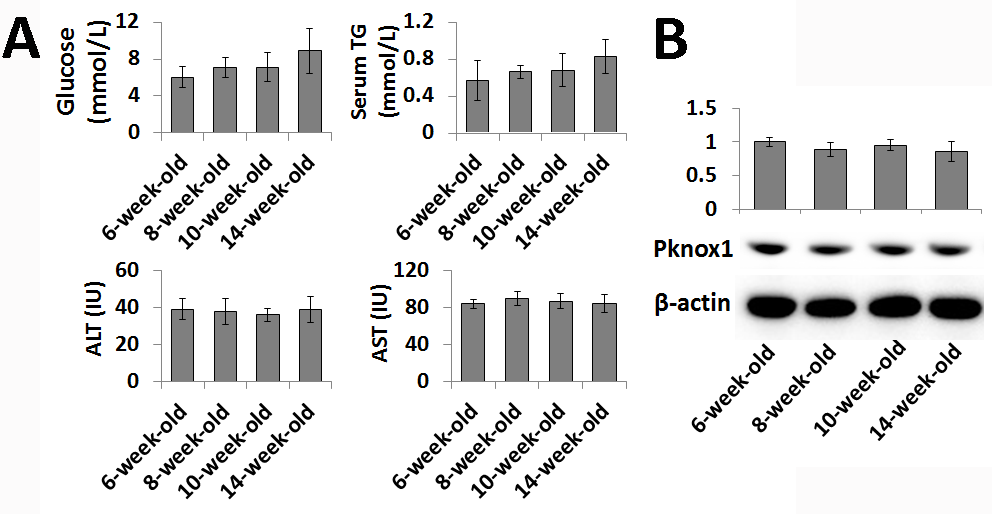
**

**Supplemental Fig. 1** **Metabolic parameters and Pknox1 expressions in nromal control rats**

(A) Blood GLU and serum TG, ALT, and AST levels were not significantly elevated at different ages of the age-matched control rats. (B) Hepatic Pknox1 levels had no differences in control rats at different ages.


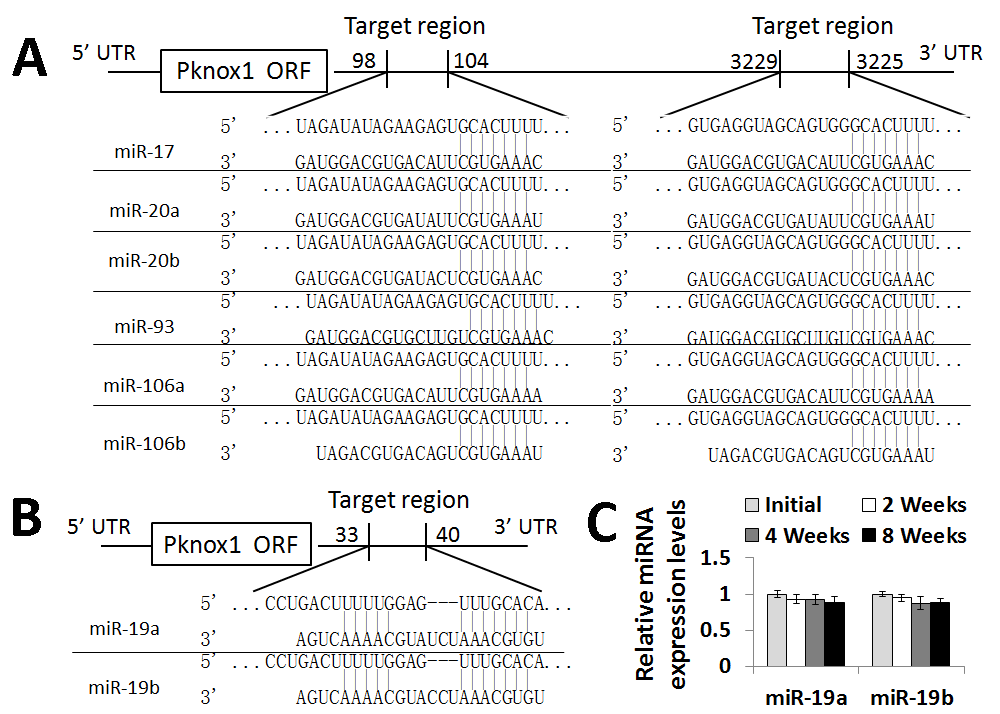


**Supplemental Fig. 2 The 3’-UTR of Pknox1 mRNA contains miR-17-92 cluster binding sites**

(A and B) Predicted-binding sites of miR-17 (A) and miR-19 (B) seed families in the 3’-UTR of Pknox1 mRNA. (C) The hepatic expression levels of miR-19a and miR-19b had no alteration in the rat model of T2DM complicated with NAFLD. Data are presented as the mean ± S.D. (**P* < 0.05, ***P* < 0.01, n = 3).

**
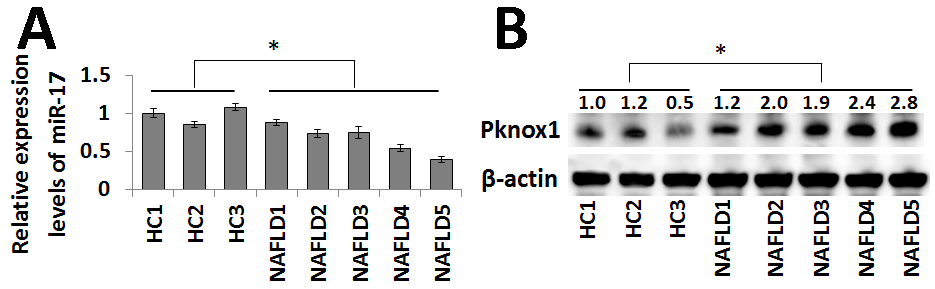
**

**Supplemental Fig.3 Expression levels of miR-17 and Pknox1 in human liver samples**

(A) The relative expression levels of miR-17 in human liver samples were detected by qPCR and analyzed by 2^-ΔΔCT^ method. MiR-17 relative levels were normalized over the average CT value of U6. (B) The protein levels of Pknox1 in human samples were determined by Western blot analysis. Grayscale ratios of the protein bands were normalized first with β-actin and then with the ratios of HC1 sample. HC, healthy control; NAFLD, non-alcoholic fatty liver disease; NAFLD4 and NAFLD5 are complicated with T2DM; **P* < 0.05.


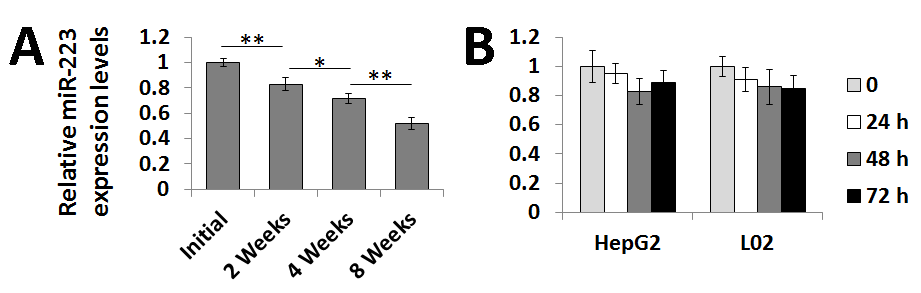


**Supplemental Fig. 4 Hepatic and cellular expression levels of miR-223**

(A) The hepatic expression levels of miR-223 were gradually decreased in a time-dependent manner in the rat model of T2DM complicated with NAFLD. (B) The relative miR-223 expression levels in HepG2 and L02 cells were not significantly changed after FFA exposure. Data are presented as the mean ± S.D. (**P* < 0.05, ***P* < 0.01, n = 3).
